# Non-adherence to community oral-antibiotic treatment in children with fast-breathing pneumonia in Malawi– secondary analysis of a prospective cohort study

**DOI:** 10.1186/s41479-016-0024-8

**Published:** 2016-11-24

**Authors:** Rebecca Nightingale, Tim Colbourn, David Mukanga, Limangeni Mankhambo, Norman Lufesi, Eric D. McCollum, Carina King

**Affiliations:** 1 Institute for Global Health, University College London, London, UK; 2 Science and Health Impact Group (SHI), Kampala, Uganda; 3 Parent and Child Health Initiative, Lilongwe, Malawi; 4 Acute Respiratory Infection Unit, Ministry of Health, Lilongwe, Malawi; 5 Department of Pediatrics, Division of Pulmonology, Johns Hopkins School of Medicine, Baltimore, USA

**Keywords:** Pneumonia, Non-adherence, Oral antibiotics, iCCM, Child, Sub-Saharan Africa, Treatment failure

## Abstract

**Background:**

Despite significant progress, pneumonia is still the leading cause of infectious deaths in children under five years of age. Poor adherence to antibiotics has been associated with treatment failure in World Health Organisation (WHO) defined clinical pneumonia; therefore, improving adherence could improve outcomes in children with fast-breathing pneumonia. We examined clinical factors that may affect adherence to oral antibiotics in children in the community setting in Malawi.

**Methods:**

We conducted a sub-analysis of a prospective cohort of children aged 2–59 months diagnosed by community health workers (CHW) in rural Malawi with WHO fast-breathing pneumonia. Clinical factors identified during CHW diagnosis were investigated using multivariate logistic regression for association with non-adherence, including concurrent diagnoses and treatments. Adherence was measured at both 80% and 100% completion of prescribed oral antibiotics.

**Results:**

Eight hundred thirty-four children were included in our analysis, of which 9.5% and 20.0% were non-adherent at 80% and 100% of treatment completion, respectively. A concurrent infectious diagnosis (OR: 1.76, 95% CI: 0.84–2.96/OR: 1.81, 95% CI: 1.21–2.71) and an illness duration of >24 h prior to diagnosis (OR: 2.14, 95% CI: 1.27–3.60/OR: 1.88, 95% CI: 1.29–2.73) had higher odds of non-adherence when measured at both 80% and 100%. Older age was associated with lower odds of non-adherence when measured at 80% (OR: 0.41, 95% CI: 0.21–0.78).

**Conclusion:**

Non-adherence to oral antibiotics was not uncommon in this rural sub-Saharan African setting. As multiple diagnoses by the CHW and longer illness were important factors, this provides an opportunity for further investigation into targeted interventions and refinement of referral guidelines at the community level. Further research into the behavioural drivers of non-adherence within this setting is needed.

## Background

Pneumonia is the second leading cause of death among children under five years of age and the leading cause of death from an infectious disease, accounting for nearly 1 million deaths annually, with the greatest burden of disease falling in Africa [1, 2]. The World Health Organisation (WHO) recommends the use of integrated community case management (iCCM) at the community health worker (CHW) level [3]. These guidelines categorise pneumonia into non-severe pneumonia or ‘fast-breathing pneumonia’ (presence of a cough and/or difficult breathing with a fast respiratory rate for age) and advise treatment with oral antibiotics in the community, or severe pneumonia (presence of cough and/or difficult breathing with chest in-drawing or danger signs, irrespective of an increased respiratory rate for age), which requires referral to a hospital [4]. Treatment failure in those who have sought healthcare within the community setting may contribute to the high mortality rate from pneumonia in developing countries [5]. A recent systematic review reported treatment failure rates in fast-breathing pneumonia ranging between 7.8–22.9% [5], with a range of possible causes including incorrect diagnosis, host comorbidities, concurrent diagnoses, antibiotic resistance, and non-adherence to oral antibiotics [6–8].

The development of tools to predict treatment failure have tended to focus on clinical presentation; however, with evidence demonstrating that non-adherence increases treatment failure only [6], it stands to reason that if adherence to oral antibiotics in community treated pneumonia were improved, treatment failure rates could be reduced. Therefore, approaches that aim to improve adherence to oral antibiotics should be considered.

There are few studies that relate to short-term medication use in children that report a vast range of non-adherence, from 3–60% [9–12]. To our knowledge, there are no published articles with a primary outcome of factors affecting adherence in children taking oral antibiotics in sub-Saharan Africa. Several publications have described different aspects of adherence related to chronic conditions in sub-Saharan Africa and acute conditions within the developed world. There is substantial evidence that prescribing ‘patient friendly drugs’—for example, reducing the duration of a course or number of tablets per day—improved adherence [8, 13–17]. When treating children in sub-Saharan Africa diagnosed with pneumonia, a three-day course of amoxicillin has been shown to be as effective as a five-day course, and improved adherence rates from 85% to 94% [18]. Other factors that were shown to have some influence on adherence included therapy-related reasons (such as taste and texture) [11]; a patient’s relationship with the health provider; access to healthcare; socioeconomic status; and condition-related factors (e.g. how unwell the patient is) [13, 19]. However, there is limited and conflicting evidence surrounding all these factors, particularly in acute conditions such as pneumonia, with a synthesis of systematic reviews finding over 770 separate factors in 51 systematic reviews of long-term treatments, yet found no publication focusing on short-term treatments [13].

In light of this evidence gap, we aimed to investigate clinical and diagnostic factors associated with non-adherence to oral antibiotic treatment of fast-breathing pneumonia in children at the community level, using data from a prospective cohort in rural Malawi.

## Method

This study is a sub-analysis of data collected during a prospective cohort, between September 2013 and June 2014, of children treated with oral co-trimoxazole for community-treated iCCM pneumonia in Mchinji District, Malawi.

### Data collection

The full methods of data collection have previously been published [6]. Briefly, the study population comprised all children aged 2–59 months diagnosed with fast-breathing pneumonia by 34 government-employed CHWs (called Health Surveillance Assistants) at their village clinics in rural Mchinji and Lilongwe districts, central region, Malawi. All children were dispensed oral co-trimoxazole to take in the community. All eligible participants who presented to CHWs and consented were recruited. Data were collected from 18 clinics in Mchinji and 16 in rural Lilongwe. Diagnoses and clinical assessments, including pulse oximetry, were recorded by CHWs on case report forms. These forms were barcoded, checked by supervisors, entered into a database, and regularly cleaned throughout the project. Lay village-level data collectors were recruited from the local area and received one week’s training on the project protocol and clinical measurements. They conducted interviews at day 5 and day 14 in the home setting, with day 0 being the day of diagnosis. When subjects were not at home on day 5, re-visits were made up to day 7. At these follow-up visits the data collectors repeated a clinical examination for pneumonia, visually confirmed reported antibiotic adherence with pill counts, and asked about further care seeking and treatments.

#### Definitions

*Community treated iCCM pneumonia* was defined as as the observation or caregiver report of cough and/or difficult breathing and observed fast breathing (>50 breaths per minute for infants 2–11 months; >40 breaths per minute for children 12–59 months), in the absence of any danger signs or chest in-drawing [20]. Danger signs included vomiting everything, inability to feed, convulsions, sleepiness or unconsciousness, and signs of severe respiratory distress including grunting, oxygen saturation <90%, nasal flaring and head nodding. These were assessed based on the caregivers report and clinical assessment by the village data collectors or CHWs.

*Non-adherence* was measured using pill counts at the day 5 visit. For the purpose of comparison we defined non-adherence as both those who completed less than 80% (i.e. 7 or less of the 10 doses) and those who completed less than 100% (i.e. missed any dose) of the 5-day, twice-daily course of co-trimoxazole. This regime is the same as for amoxicillin and therefore the definition of non-adherence can be applied as countries transition from co-trimoxazole to amoxicillin. As per the standard Malawi guidelines one dose is ½ tablet for 2–11 months and 1 tablet 12–59 months.

*Treatment failure* was defined as the presence of any of the following on the day after the final dose of antibiotics: fast breathing for age, axillary temperature >37.5 °C, lower chest in-drawing, any danger sign, change of antibiotic, hospital admission or death.

### Analysis

Data were analysed using Stata version 13.1 statistical software. We described non-adherence and conducted multivariate analysis using logistic regression with 80% and 100% adherence as the outcome variables in separate models. Based on the literature and prior knowledge, we included clinical presentation and concurrent diagnoses and treatments in the multivariate model. Clinical presentation was included using the following variables: temperature (normal: <=37.4 °C, fever: > 37.4 °C); oxygen saturation (normal = > 95%, abnormal <95%); age-adjusted respiratory rate as breaths per minute (fast: 40–59 [age 12–59 months] 50–69 [age 2–11 months], very fast: > = 60 [age 12–59 months] > =70 [age 2–11 months]); and heart rate as beats per minute (normal: <159 [age 2–11 months] <149 [age 12–23 months] <139 [age 24–59 months]). Concurrent diagnoses at presentation included: presumptive malaria (not laboratory or rapid diagnostic test confirmed), diarrhoea; ear infection, rash and other specified infection. Concurrent medications included: Paracetamol, Lumefantrine Artemether (LA) and others specified (including aspirin, topical creams, oral salbutamol). Duration of illness prior to seeking care (caregiver reported as over 24 h or under 24 h), child vomiting after antibiotics, age (2–12 months, 1–2 years and 2–5 years) and gender were also included.

To determine the inclusion of individual or composite formulations of medication and concurrent diagnosis variables, we looked at the correlation between these individual and composite variables; variables that had a correlation of above 0.7 were not included in the same model [21]. This resulted in the composite diagnosis/medication variables being analysed separately from the individual diagnosis and medication variables. The overall percentage of missing data was low, with all variables having less than 5% missing data, and overall 12% of records with any missing data. A complete case analysis approach was taken.

## Results

A total of 974 cases were presented for analysis. Of these, 23 did not meet the criteria of fast-breathing pneumonia (had no cough or difficulty breathing), and 117 were not at home on day 5 when the pill count was completed. These patients were not included for analysis, which resulted in a total of 834 cases being analysed.

Table 1 summarises the baseline characteristics of the sample population and description of the clinical variables. Previously published baseline variables show those lost to follow up had lower rates of malnutrition but also lower rates of uptake of the PCV vaccination [6]. The percentage of children diagnosed with another condition concurrently to pneumonia was high (50.7%). Malaria was the most common concurrent diagnosis, occurring in 41.4% of children, and 61.8% of children received any medication in addition to oral co-trimoxazole. Caregiver histories revealed that 58% of children were brought to the clinic for assessment on the day they became unwell or within 24 h of illness onset. Just over 12% of children vomited after taking any dose of co-trimoxazole.

**Table 1:** Characteristics and description of the study population

*N* = 834			
		N (%)	
Age	2–11 months	268 (32.1%)	
	12–23 months	273 (32.7%)	
	24–59 months	293 (35.1%)	
Gender	Female	429 (51.4%)	
	Male	391 (46.9%)	
	Missing	14 (1.7%)	
District	Kabudula	479 (57.4%)	
	Mchinji	355 (42.6%)	
Clinical malaria diagnosis	Yes	345 (41.4%)	
	No	489 (58.6%)	
Diarrhoea diagnosis	Yes	28 (3.4%)	
	No	806 (96.6%)	
Any concurrent diagnosis^a^	Yes	423 (50.7%)	
	No	411 (49.3%)	
Prescribed LA^b^	Yes	430 (51.6%)	
	No	404 (48.4%)	
Prescribed paracetamol	Yes	430 (51.6%)	
	No	404 (48.4%)	
Prescribed multiple drugs	Yes	515 (38.2%)	
	No	319 (38.3%)	
Duration of illness before diagnosis	<24 h	482 (58.1%)	
	>24 h	347 (41.9%)	
Vomited after taking antibiotics	Yes	726 (12.7%)	
	No	106 (87.3%)	
		Number	Median^c^ (range)
Respiratory rate (breaths/min)	Age 2–11 mths	253	55 (40–83)
	Age 12–59 mths	545	46 (40–100)
Temperature (°C)		803	42.0 (34.3–42)
Oxygen Saturation (%)		826	97 (90–100)
		Number	Mean^c^ (SD)
Heart rate (beats/min)	Age 2–11 mths	260	136 (24.7)
	Age 12–23 mths	260	138 (24.9)
	Age 24–59 mths	279	129 (23.2)

*LA* lumefantrine artemether, *SD* standard deviation

^a^Diagnoses included: malaria, diarrhoea, ear infection, rash and other specified

^b^The child was presumptively prescribed the anti-malaria Lumefantrine Artemether (LA) by the community health worker

^c^Median used for skewed data, mean for normally distributed data

Non-adherence to oral co-trimoxazole was 9.5% when measured at 80%, and 20.0% at 100% completion. Treatment failure at day 5 was 14.9%, and 7.0% at day 14. While our aim was not to investigate treatment failure, the percentage of children failing treatment was slightly higher in those that were non-adherent (19.0%; 95% CI: 16.3–22.0) versus adherent (14.4%; 95% CI: 12.0–17.1); however, this study was not powered to look at this association. The results of the multivariate model are presented in Table 2 (bivariate analysis is available in the Appendix). For non-adherence measured at both 80% and 100%, children with any concurrent diagnosis had higher odds of being non-adherent to medication (OR: 1.76, 95% CI: 1.00–3.07/OR: 1.81 95% CI: 1.21–2.71, respectively). In the multivariate model, concurrent malaria diagnosis alone was not significantly associated with non-adherence (OR: 1.20; 95% CI: 0.67–2.15/OR: 1.41 95% CI: 0.93–2.17). Children who were unwell for more than 24 h before CHW diagnosis had significantly increased odds of non-adherence at 80% and 100% (OR: 2.14, 95% CI: 1.27–3.58/OR: 1.88, 95% CI: 1.29–2.73). Being in the older age group was associated with lower odds of <80% adherence (OR: 0.41, 95% CI: 0.21–0.78), but this was not the case for <100% adherence. Children prescribed multiple medications showed increased odds of non-adherence in bivariate analysis, but not in the adjusted analysis. The clinical severity of the child’s pneumonia at diagnosis did not show a significant relationship with non-adherence, nor did a child vomiting after their antibiotics.

**Table 2:** Multivariable regression for clinical and diagnostic factors associated with non- adherence

	Adherence measured at 80%			Adherence measured at 100%		
Variable	Odds Ratio	P Value	CI	Odds ratio	P Value	CI
Any Concurrent Diagnosis^a^	1.75	0.050	1.00–3.07	1.81	0.004	1.21–2.71
Prescribed multiple drugs^b^	1.57	0.159	0.84–2.96	1.09	0.711	0.70–1.68
Duration of illness > 24 h	2.14	0.004	1.27–3.58	1.88	0.001	1.29–2.73
Abnormal oxygen saturation^c^	0.82	0.606	0.39–1.73	1.44	0.148	0.88–2.36
Very fast breathing^d^	0.82	0.754	0.22–2.92	0.66	0.355	0.27–1.60
Abnormal heart rate^e^	0.80	0.419	0.41–1.45	1.41	0.123	0.91–2.17
Fever^f^	1.50	0.150	0.86–2.60	1.06	0.777	0.70–1.61
Vomited antibiotics	0.82	0.605	0.38–1.77	0.85	0.574	0.48–1.50
Age (12–23months)	0.68	0.206	0.38–1.23	1.05	0.822	0.67–1.65
Age (24–56 month)	0.41	0.007	0.21–0.78	0.68	0.111	0.43–1.09
Sex (Male)	0.91	0.740	0.54–1.51	1.06	0.750	0.73–1.54
Pseudo R2 = 0.061				Pseudo R2 = 0.045		

^a^Diagnoses included: malaria, diarrhoea, ear infection, rash and other specified

^b^Drugs included: Paracetamol, Lumefantrine Artemether (LA) and others specified (including aspirin, topical creams, oral salbutamol)

^c^Normal: = > 95%; Abnormal: <= 94%

^d^Fast breathing (breaths per minute): 40–59 (age 12–59 months) 50–69 (age 2–11 months); Very fast breathing: > = 60 (age 12–59 months) > =70 (age 2–11 months)

^e^Normal (beat per minute): <159 (age 2-11months), <149 (age 12–23 months), <139 (age 24–59 months); Abnormal: > = 159 (age 2-11months), > = 149 (age 12–23 months), > = 139 (age 24–59 months)

^f^Normal: <=37.4 °C; Fever: > 37.4 °C

## Discussion

We sought to describe the effect of clinical presentation on adherence to oral antibiotics in children diagnosed with community-treated iCCM pneumonia in rural Malawi. Our results highlighted that there is a level of non-adherence to oral antibiotics within these communities, with 20% of children not finishing their full course of antibiotics and nearly 10% not completing 80% of the course. There is limited data to compare this to, with available literature reporting a vast range of non-adherence from 3–60% [9–12]. Having a secondary diagnosis and presenting for treatment having been unwell for more than 24 h before diagnosis were associated with non-adherence amongst this population.

### Clinical severity

The limited literature available suggests that the more seriously sick a child is, the more likely they are to be adherent to medication [22]; however, our results did not find severity to be a main driver of adherence in this group. It is plausible that the way this study has measured clinical presentation is not how a caregiver would assess it, since measures such as oxygen saturation are non-visible whereas a fever or faster respiratory rate may be visible to a caregiver. As these children were being treated in the community, caregivers may not have perceived them as seriously unwell. This is supported by the fact that they were seeking care in the community. However, with no studies reviewing children with pneumonia in an African setting, it is possible that the different cultural and local health beliefs mean severity and risk are viewed differently, giving alternative results. Evidence suggests that in Malawi, especially in areas with endemic tropical disease, health beliefs vary greatly from Western models, particularly when understanding the risk of disease [23]. There is also potential that parents did not see a benefit in continuing to give their child medication if the child (potentially with viral infections) improved or, alternatively, caregivers saw no to minimal improvement after completing 3 or 4 days of treatment. Research aiming to predict pneumonia treatment failure in Malawi showed limited success in using clinical severity as a possible predictor for failing treatment at the community level [6].

### Concurrent diagnosis and medication

Children with a concurrent diagnosis were less likely to be adherent to oral antibiotics. In this study, a large proportion of children had malaria as a secondary diagnosis, yet concurrent malaria alone does not appear to be the reason for non-adherence. Due to a lack of evidence surrounding this topic, we speculate about possible reasons. It is possible that once the child recovered from the concurrent disease, their symptoms were much better and therefore the caregiver stopped giving the antibiotics. Alternatively, as the course of medication for malaria and many other diseases are shorter than 5 days, all medications may have been stopped together. Where a caregiver receives instructions for treatment of multiple conditions, confusion and lack of understanding could result in errors following the correct prescription. It would be expected that concurrent medications and concurrent diagnosis would have almost 100% correlation, yet this was not the case. Although moderate correlation existed, the relationship was not as strong as expected (correlation = 0.50). This could be attributed to an inaccurate caregiver reporting, as well as poor implementation of the iCCM guidelines by CHWs.

At the time of this study, CHWs in Malawi were not using rapid diagnostic testing for malaria. It is well documented that over-prescription of medication, particularly in non-confirmed malaria, is a common problem in sub-Saharan Africa [24]. It is also recognised that where rapid malaria tests are available, there is a reduction in prescription of malaria medication [25]. A limitation of this work is that human immunodeficiency virus (HIV) status of children was not collected due to practical and ethical considerations because pediatric HIV testing was not widely performed by government-sector CHWs in Malawi at the time of this study [6]. Co-trimoxazole is used as a prophylactic drug in children with HIV. HIV prevalence is approximately 8% amongst adults, and therefore a substantial percentage of these children could already be taking co-trimoxazole [26]. Beliefs about the drug itself, which parents could associate with a diagnosis of HIV, may affect adherence.

### Duration of illness

Children whose caregivers reported that they had symptoms for more than 24 h were less likely to complete the course of antibiotics. We are not aware of any other comparable literature. Caregivers who took longer to present their unwell child to clinic may have experienced more problems with accessing the clinic, or may have a lower socioeconomic status with limited ability to ensure children took the medication. Similarly, a caregiver’s health beliefs may influence the length of time taken to present to the clinic or result in a perception their child was not as sick. These reasons have been shown to increase non-adherence in other conditions and could be causes of non-adherence in those who have been unwell for longer [13].

### Age

Older age was associated with improved adherence when measured at 80%, but not 100%. Co-trimoxazole prescribed in Malawi is a tablet that has to be split and crushed for young children. Evidence suggests that ‘patient friendly’ medication tends to improve adherence, particularly taste and texture with children [11]. Giving crushed tablets to a young baby is potentially not ‘patient friendly’ and difficult for the caregiver. Similar results have been seen in other studies, and suggest that alternative treatment types (e.g. syrups or dispersible tablets) should be investigated for improved adherence [13].

### Treatment failure and non-adherence

This study was not powered to determine if non-adherence was statistically associated to treatment failure. The slight increase in treatment failure in those who were non-adherent needs further investigation with an appropriately powered study. Furthermore the limitation of WHO guidelines should be taken into account, since infections treated with antibiotics in this study may have been viral and thus reasonably be expected to have improved by day 5 or 14 without treatment. There is a potential tension when refining guidelines between encouraging non-specific antibiotic prescription for possible bacterial infections (where adherence will be beneficial and shorter-term mortality benefits will be maximized) and reducing unnecessary antibiotic use for self-resolving viral infections largely responsible for the development of increasing antimicrobial resistance rates, a longer term public health concern.

### Limitations

This study was limited by the lack of socioeconomic data, with further research needed on the drivers of non-adherence from a socioeconomic and behavioural stand point. Records of a concurrent diagnosis and medication and duration of illness were from caregiver reports, where answers could be influenced by a range of socioeconomic and social desirability issues, which could lead to both over- and under-reporting. Measuring adherence by pill count assumes that the child swallowed the tablets that are not left, but it is possible they were thrown away, misplaced or lost, or sold. Over-reporting of adherence is therefore possible when using pill count as the measure [19]. Caution should also be given to the clinical measurement used in this study, there is consistent evidence that even with monthly supportive supervision and annual refresher trainings, that accurate assessment of clinical signs is poor in the community in Malawi [27, 28]. This is a significant limitation of establishing clinical severity effects on non-adherence within a community setting.

## Conclusions

This analysis suggests that non-adherence to oral antibiotics is an issue in children diagnosed with community treated iCCM pneumonia in Malawi. Although different clinical severity (mild–moderate) at diagnosis is not associated with adherence, concurrent diagnoses, treatments and duration of illness could be important factors in driving treatment decisions and behaviour. Treatment failure was higher in non-adherent children, despite not demonstrating a statistically significant association, and therefore improving adherence is a potential area for improved community case management of pneumonia. As multiple diagnoses and treatments were highlighted as risks for poor adherence, refined diagnosis and treatment pathways in these potentially clinically complex children needs more investigation. Further research is required into the drivers of treatment decisions by caregivers and CHWs for acute infections in a sub-Saharan African community setting to inform this process, and to fill a considerable evidence gap.

## Appendix

**Table 3:** Bivariate analysis of clinical and diagnostic factors associated with non-adherence at 80% and 100% completion

		80% adherence					100% adherence				
		Adherent	Non-adherent	OR	*p*-value	95% CI	Adherent	Non-adherent	OR	*p*-value	95% CI
Fever^a^	Normal	493	45				432	106			
	High	234	31	1.45	0.129	0.89, 2.36	208	57	1.12	0.549	0.78, 1.60
Oxygen Saturation^b^	Normal	643	66				566	134			
	Abnormal	113	13	1.11	0.754	0.59, 2.07	93	33	1.50	0.070	0.96, 2.33
Respiratory Rate^c^	High	680	75				599	156			
	V.High	40	3	0.68	0.526	0.21, 2.25	35	8	0.88	0.745	0.40, 1.93
Heart Rate^d^	Normal	541	60				477	112			
	High	182	16	0.79	0.429	0.45, 1.41	162	48	1.26	0.232	0.86, 1.85
Clinical Malaria	No	453	36				408	81			
	Yes	302	43	1.79	0.013	1.12, 2.86	259	86	1.67	0.003	1.19, 2.36
Diarrhoea	No	733	73				648	158			
	Yes	22	6	2.74	0.028	1.07, 6.99	19	9	1.94	0.103	0.86, 4.38
Any Concurrent Diagnosis	No	397	26				361	62			
	Yes	358	53	2.26	0.001	1.38, 9.71	306	105	2.00	0.001	1.40, 2.82
LA^e^	No	377	27				340	64			
	Yes	378	52	1.92	0.008	1.80, 3.13	327	103	1.67	0.004	1.18, 2.37
Paracetamol	No	381	23				342	62			
	Yes	374	56	2.48	0.001	1.49, 4.13	325	105	1.78	0.001	1.25, 2.53
Multiple Drugs	No	300	19				269	50			
	Yes	455	60	2.08	0.006	1.21, 3.57	398	117	1.58	0.013	1.10, 2.28
Duration of illness	<24h	450	32				408	74			
	>24h	301	46	2.15	0.001	1.33, 3.46	255	92	1.99	0.001	1.41, 2.82
Vomited after antibiotics	No	658	68				581	145			
	Yes	96	10	1.01	0.982	0.50, 2.03	85	21	0.99	0.969	0.59, 1.65
Age	2–11mths	233	35				213	55			
	12–23mths	247	26	0.70	0.195	0.41, 1.20	210	63	1.16	0.472	0.77, 1.75
	24–59mths	275	18	0.44	0.006	0.24, 0.79	244	49	0.79	0.248	0.52, 1.21
Sex	Male	355	36				311	80			
	Female	386	43	0.91	0.693	0.57, 1.45	342	87	1.01	0.949	0.72, 1.42

^a^Normal: <=37.4 °C, High: > 37.4 °C ; ^b^Normal: = > 95% Abnormal: <= 94%; ^c^High: 40–59 (age 12–59 months) 50–69 (age 2–11 months), Very High: > = 60 (age 12–59 months) > =70 (age 2–11 months); ^d^Normal: <159 (age 2–11months), <149 (age 12–23 months), <139 (age 24–59 months). LA: Lumefantrine Artemether

^e^Lumefantrine Artemether prescribed by the community health worker
